# Evaluation of MODIS-derived estimates of the albedo over the Atacama Desert using ground-based spectral measurements

**DOI:** 10.1038/s41598-021-98622-4

**Published:** 2021-10-06

**Authors:** Raúl R. Cordero, Sarah Feron, Edgardo Sepúlveda, Alessandro Damiani, Juan M. Carrera, Jose Jorquera, Juan A. Alfonso, Rosalino Fuenzalida, Miguel Rivas, Shelley MacDonell, Gunther Seckmeyer, Chenghao Wang, Zutao Ouyang, Stef Lhermitte

**Affiliations:** 1 Universidad de Santiago de Chile, Av. Bernardo O’Higgins, 3363 Santiago, Chile; 2 Department of Earth System Science, Stanford University, Stanford, CA 94305 USA; 3 Center for Environmental Remote Sensing, Chiba University, Inage Ward, 1-33 Yayoicho, Chiba, 263-8522 Japan; 4 Instituto Venezolano de Investigaciones Cientificas (IVIC), Apartado, Caracas, 20632 Venezuela; 5 Universidad Arturo Prat, Avenida Arturo Prat 2120, Casilla 121, Iquique, Chile; 6 Universidad de Tarapacá, Avenida General Velásquez, 1775 Arica, Chile; 7 Centro de Estudios Avanzados en Zonas Aridas (CEAZA), La Serena, Chile; 8 Leibniz Universität Hannover, Herrenhauser Strasse 2, Hannover, Germany; 9 Department of Geoscience and Remote Sensing, Delft University of Technology, Delft, The Netherlands; 10 University of Groningen, Wirdumerdijk 34, 8911 CE Leeuwarden, Netherlands

**Keywords:** Atmospheric science, Solar energy

## Abstract

Surface albedo is an important forcing parameter that drives the radiative energy budget as it determines the fraction of the downwelling solar irradiance that the surface reflects. Here we report on ground-based measurements of the spectral albedo (350–2200 nm) carried out at 20 sites across a North–South transect of approximately 1300 km in the Atacama Desert, from latitude 18° S to latitude 30° S. These spectral measurements were used to evaluate remote sensing estimates of the albedo derived from the Moderate Resolution Imaging Spectroradiometer (MODIS). We found that the relative mean bias error (*RMBE*) of MODIS-derived estimates was within ± 5% of ground-based measurements in most of the Atacama Desert (18–27° S). Although the correlation between MODIS-derived estimates and ground-based measurements remained relatively high (R= 0.94), *RMBE* values were slightly larger in the southernmost part of the desert (27–30° S). Both MODIS-derived data and ground-based measurements show that the albedo at some bright spots in the Atacama Desert may be high enough (up to 0.25 in visible range) for considerably boosting the performance of bifacial photovoltaic technologies (6–12%).

## Introduction

The surface albedo is defined as the ratio of surface-reflected irradiance to incident irradiance over a given spectral interval^1–4^. The albedo varies temporally and spatially as a result of natural processes (such as vegetation growth and snowfall) and anthropogenic activities (deforestation/forestation, agriculture, wildfires, etc.). Relative to forests and vegetation, snow-covered surfaces and dusty deserts exhibit higher albedo. However, there are substantial spectral differences; while the albedo of snow-covered surfaces maximizes in the visible part of the spectrum^1–3^, the albedo of dusty deserts reaches its peaks in the near-infrared (NIR) part of the spectrum^4^.

The surface albedo is an important forcing parameter that drives the radiative energy budget as it determines the fraction of the downwelling solar irradiance that the surface reflects. Therefore, changes in the planetary albedo resulting from the extensive land use change^5,6^ or from precipitation regime anomalies^7^ are of great interest. The relatively high albedo of arid and semi-arid regions is also drawing attention because of its contribution to the solar energy potential. At low- and mid-latitudes, regions of high albedo (arid and semi-arid territories) are likewise regions of great solar energy potential^8^.

The albedo contributes to the solar photovoltaic (PV) potential as multiple scatterings between the surface and the atmosphere enhance the downwelling irradiance^3^. Moreover, the surface-reflected radiation flux can be used to directly generate electric power by using bifacial solar PV modules^9,10^. These transparent-backsheeted modules expose their front and backside to the downwelling and the upwelling irradiance, respectively, increasing the energy yield per square meter of PV module^11,12^. The solar potential of the Atacama Desert in particular is likely the largest on Earth^13,14^.

**Figure 1:**
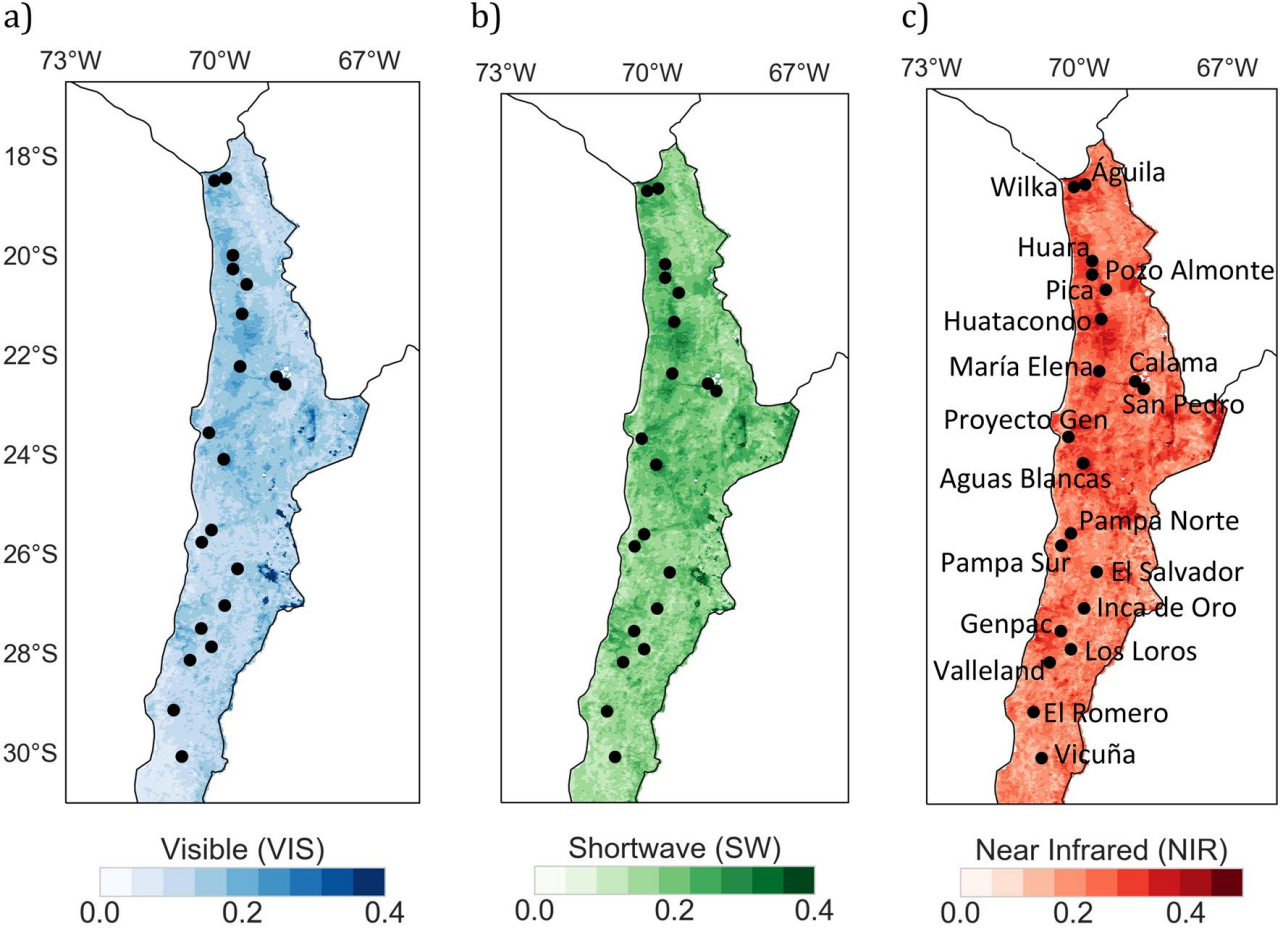
Satellite-derived estimates of the white-sky albedo averaged over the period 2011–2020 for the following broadbands: (**a**) Visible (VIS), (**b**) Shortwave (SW), and (**c**) Near infrared (NIR). Measurement sites are also indicated in the plots. Plots were generated using Python’s Matplotlib library^44^ with data from the Moderate Resolution Imaging Spectroradiometer (MODIS) MCD43A3 Version 6 Albedo Model dataset^24^.

Although satellite-derived estimates show that the albedo of the Atacama Desert is relatively high (Fig. 1), albedo-related satellite products over the region have not been subjected to validation efforts by using ground-based measurements. Here, we report on ground-based measurements of the spectral albedo (350–2200 nm) carried out in September 2018 at twenty sites in the Atacama Desert (18–28° S) (Fig. 1); elevation ranged from 485 to 2583 m above mean sea level (AMSL) (see Table 1). These spectral measurements were used to evaluate remote sensing estimates of the albedo derived from the Moderate Resolution Imaging Spectroradiometer (MODIS)^15–17^ instrument. MODIS-derived estimates enabled further comparisons between the albedo of the Atacama Desert and the albedo of other arid and semi-arid regions around the world.

**Table 1:** Measurement sites. “SZA” refers to the solar zenith angle at the moment of the measurement while “distance” refers to the distance between the measurement site and the center of the closest MODIS data grid.

Site	Latitude (º)	Longitude (º)	Date (dd/mm)	Time (UTC-3) (hh:mm)	SZA (^o^)	Elevation (m)	Distance (m)
Águila	− 18.4429	− 69.8929	29/09	16:10	42.2	1655	153
Wilka	− 18.4937	− 70.1126	29/09	14:00	17.4	907	260
Huara	− 19.9937	− 69.7528	30/09	11:50	29.7	1111	3370
Pozo Almonte	− 20.2655	− 69.7508	30/09	12:30	22.6	1031	240
Pica	− 20.5668	− 69.4733	30/09	13:30	17.6	1042	282
Huatacondo	− 21.1721	− 69.5597	30/09	14:35	24.3	789	100
María Elena	− 22.2207	− 69.5965	28/09	14:00	21.4	1164	238
Calama	− 22.4302	− 68.8725	28/09	12:20	25.9	2357	182
San Pedro	− 22.5762	− 68.7030	28/09	11:25	35.7	2583	220
Proyecto Gen	− 23.5643	− 70.2297	27/09	15:55	40.8	575	100
Aguas Blancas	− 24.0897	− 69.9284	27/09	11:45	34.0	968	166
Pampa Norte	− 25.5121	− 70.1761	26/09	16:05	43.9	1250	253
Pampa Sur	− 25.7451	− 70.3683	26/09	15:00	32.1	888	213
El Salvador	− 26.2864	− 69.6482	26/09	12:05	32.3	2059	235
Inca de Oro	− 27.0346	− 69.9107	25/09	14:40	30.7	1696	234
Genpac	− 27.4886	− 70.3798	25/09	13:25	26.6	766	272
Los Loros	− 27.8618	− 70.1832	25/09	11:40	38.1	1560	201
Valleland	− 28.1214	− 70.6103	24/09	14:20	29.5	485	249
El Romero	− 29.1274	− 70.9282	24/09	12:45	31.0	1150	177
Vicuña	− 30.0617	− 70.7660	23/09	15:30	40.3	640	138

## The Atacama Desert

The Atacama Desert is a 1300 km-long (18–30° S) narrow strip between the Andes to the east and the Pacific Ocean to the west. The Andes Mountains act as a barrier to moisture transport from the Amazon basin leading to an intense rain shadow effect, while the cold-water current of the southeast Pacific Ocean chills the air limiting the amount of moisture it can hold^18,19^.

In the northern part of the Atacama Desert, the South Pacific Anticyclonic (SPA) circulation blocks storms from the Pacific moving into the area. Although the South American monsoon system^20,21^ enhances moisture transport from the Amazon basin, which leads to orographic rainfall during the austral summer^22,23^, precipitations are normally constrained to the mountains. With some areas that receive just a few millimeters of rain per year and some that see none at all, the northern Atacama Desert is one of the driest places in the world. In the southern part of the Atacama Desert, precipitations are less scarce (in the range of tens of millimeters per year) and are not restricted to the mountains. Rainfall in this area is associated with passing frontal systems (and cut-off lows) that occur mainly during the wet season (austral fall and winter)^24^. Due to relatively higher precipitations, the landscape in the southern Atacama Desert is greener (i.e., it has more vegetation) than in the northern Atacama Desert, which in turn makes local albedo lower.

## Data

### Ground-based measurements

The surface albedo is defined as the instantaneous ratio of surface-reflected irradiance to incident irradiance over a given spectral interval (dimensionless). In our case, we computed the albedo by using measurements, taken sequentially, of the up- and down- welling global spectral irradiance. The measurements were carried out between 350 and 2200 nm (with a spectral resolution of 1 nm) by using an Analytical Spectral Devices (ASD) FieldSpec 4 hyperspectral spectroradiometer (Malvern Panalytical, USA), at twenty sites in the Atacama Desert (Fig. 1 and Table 1).

The sites selected (Figs. S1–S3) for our ground-based measurements were relatively flat, level and homogeneous (with no vegetation when possible). In order to ensure that the sites were adequately flat, we selected surfaces of at least 200 m in diameter with less than 2 degrees of level (this was controlled in situ). We also preferred sites without noticeable plant or vegetation, although this was not possible in the southernmost part of the desert (Fig. S3) where scattered vegetation is more frequent. In order to ensure a proper representativeness, sites were selected along a transect of approximately 1300 km that practically crossed the whole Atacama Desert (from latitude 18° S to latitude 30° S). We also made sure that the selected sites included both bright and dark spots (i.e., surfaces of low and high albedo). This aimed to ensure that the albedo measurements would exhibit the relatively wide dispersion suggested by MODIS-derived data for the Atacama Desert (from 0.1 to 0.25 in the visible part of the spectrum).

Each site was visited once during a campaign conducted in late September 2018. Following the methodology used elsewhere^1–4^, the rotating input optics, fitted with a flat diffuser, was set up at 70 cm above the surface and 1.5 m away from the heavy-duty tripod where the spectroradiometer was installed. Although the field-of-view (FOV) of our input optics is *nominally* 180º, prior efforts^3^ have shown that over 90% of the surface-reflected upwelling irradiance reaching the input optics corresponds to a visual angle of about 72º. Taken the latter as an *effective* FOV, we estimate that the footprint of our albedo measurements corresponds to an area slightly larger than 2 m in diameter. The operator was located several meters away to avoid shadowing. The nadir orientation was determined by practice with a level. Due to the time needed to accomplish two consecutive scans and rotate the input optics, the up- and downwelling spectra were separated in time about 3 min.

Measurements were collected in triplicate. The mean of the three measurements was calculated for each site. Quality assurance included corrections for dark signal and for cosine error of the input optics. Although they were conducted under cloudless conditions, the cosine error is expected to be low^25,26^ because the measurements were carried out relatively close to noon (when the solar elevation in the Atacama Desert is high).

Post-processing of the data involved computing spectral albedo as the ratio between the upwelling and the downwelling irradiance for each wavelength. In the shortwave infrared wavelengths, the signal-to-noise ratio was too low around the water vapor absorption bands (950 nm, 1150 nm, 1400, 1850 nm). Therefore, spectra were truncated around these bands. Although our measurements have a high spectral resolution (1 nm), that is not the case for satellite estimates; MODIS-derived data are provided for seven spectral bands ranging from the visible to the near infrared part of the spectrum (see details below). Due to the different spectral resolution of ground-based measurements and MODIS-derived data, comparisons required convolving our spectral measurements of the albedo by using the spectral response functions of the MODIS sensor (http://mcst.gsfc.nasa.gov/calibration/parameters).

### Satellite estimates

Remote sensing estimates of the albedo were obtained from the Moderate Resolution Imaging Spectroradiometer (MODIS) MCD43A3 Version 6 Albedo Model dataset^16^. MODIS is an instrument aboard the Terra and Aqua satellites. Terra's orbit around the Earth is timed so that it passes from north to south across the equator in the morning, while Aqua passes south to north over the equator in the afternoon. Terra MODIS and Aqua MODIS are viewing the entire Earth's surface every 1 to 2 days, acquiring data in 36 spectral bands.

The MCD43A3 Version 6 Albedo Model dataset is produced daily using 16 days of Terra and Aqua MODIS data at 500 m resolution. The MCD43A3 provides black-sky albedo (*A*_*n*_, directional hemispherical reflectance) and white-sky albedo (*A*_*w*_, bihemispherical reflectance) data at local solar noon for MODIS bands (Band 1 (620–670 nm), Band 2 (841–876 nm), Band 3 (459–479 nm), Band 4 (545–565 nm), Band 5 (1230–1250 nm), Band 6 (1628–1652 nm), and Band 7 (2105–2155 nm)) as well as for three broadbands: visible (VIS) (300–700 nm), near infrared (NIR) (700–5000 nm) and shortwave (SW) (300–5000 nm). The black-sky albedo and the white-sky albedo were used to compute the so-called blue-sky albedo (*A*_*b*_)^27,28^, which was in turn compared with our ground-based measurements of the albedo. The blue-sky albedo (*A*_*b*_) was calculated by applying the following equation:

1 $$A_{b} = \gamma \cdot A_{w} + ({1}{-}\gamma) \cdot A_{n} ,$$

where *γ* is the fraction of diffuse irradiance (that depends on the solar elevation) at the moment of the ground-based measurement. We obtained the *γ* values from Fig. S4, which shows the fraction of diffuse irradiance computed for different solar zenith angles. The *γ* values in Fig. S4 were computed by using the UVSPEC radiative transfer model^29^ under the typical conditions of the Atacama Desert^13,30^ for each of the MODIS bands. The UVSPEC model has been validated by systematic comparisons with ground-based measurements under cloudless conditions in other geographic regions^31,32^. The model used the DIScrete Ordinates Radiative Transfer (DISORT) solver^33^ and the extraterrestrial spectrum by Gueymard^34^.

For solar elevations higher than 25°, the blue-sky albedo has been widely used in prior efforts^27,28,35,36^ testing satellite data with ground-based measurements. However, none of these prior comparisons with ground-based measurements has included the Atacama Desert.

## Methods

Ground-based measurements of the spectral albedo were used to evaluate remote sensing estimates. The evaluation involved comparing MODIS-derived estimates of the blue-sky albedo (computed by Eq. 1) and our ground-based measurements (previously convolved with the spectral response functions for the MODIS sensor: http://mcst.gsfc.nasa.gov/calibration/parameters). The comparisons included seven MODIS bands: Band 1 (620–670 nm), Band 2 (841–876 nm), Band 3 (459–479 nm), Band 4 (545–565 nm), Band 5 (1230–1250 nm), Band 6 (1628–1652 nm), and Band 7 (2105–2155 nm).

Considering the different footprints, here we adopted spatial and temporal requirements for the comparison between MODIS data and ground-based measurements of the albedo. The measurements must have been carried out less than 300 m from the center of the closest MODIS data grid, and within a time span around noon compatible with a solar zenith angle (SZA) lower than 40°.

For each site and for each MODIS band, we computed the absolute difference (or bias error) and the relative difference (or relative bias error) between satellite-derived estimates of blue-sky albedo and our ground-based measurement. Moreover, we clustered MODIS-derived data and ground-based measurements of the albedo: according to the latitude and according to common MODIS bands. For each of these clusters, we built up scatter plots and computed the correlation coefficient (R).

Finally, we computed the correlation between the relative bias error of the MODIS-derived estimates of the blue-sky albedo (relative to the ground-based measurements of the albedo) and the SZA (at the moment of the measurement), the distance between the measurement site and the center of the closest MODIS data grid, and the latitude and elevation of the measurement site. The correlation coefficients are shown in Table S1 in the Supplementary Information.

Informed consent was obtained for publication of identifying information/images in an online open-access publication.

## Results

### Ground-based measurements

**Figure 2:**
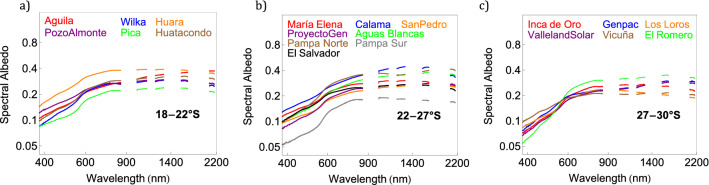
Ground-based measurements of the spectral albedo carried out at 20 sites in the Atacama Desert. Spectra are truncated around water vapor absorption bands. Sites were clustered according to the latitude: (**a**) from latitude 18° S to latitude 22° S, (**b**) from latitude 22° S to latitude 27° S, and (**c**) from latitude 27° S to latitude 30° S. Plots were generated by using Python’s Matplotlib Library^44^.

Figure 2 shows the results of our campaign aimed at measuring the spectral albedo at the 20 sites indicated in Table. 1. These measurements show that the albedo at each of the sites is relatively low in the UV part of the spectrum (wavelengths shorter than 400 nm) but increases monotonically with the wavelength within the visible range (400–700 nm) until peaking in the NIR part of the spectrum (at wavelengths in the range 1500–2000 nm). Our measurements also show a significant dispersion in the albedo that in the visible part of the spectrum ranges from about 0.1 at Pampa Sur (Fig. 2b) to about 0.25 at Huara (Fig. 2a). These differences are also apparent in dust samples taken from the surface at the measurement sites as the darker dust at Pampa Sur appreciably reflects less solar radiation than the brighter surface at Huara (Fig. S5).

The significant dispersion tends to obscure the regional differences in the albedo. However, our measurements do show that the albedo in the visible range at sites the southernmost part of the Atacama Desert (Fig. 2c) is on average about 10% lower than the albedo at sites in the northernmost section of the Atacama Desert (Fig. 2a). The regional differences considerably increase in the UV range; for wavelengths shorter than 400 nm, the albedo in the UV range at sites in the southern section of Atacama Desert (Fig. 2c) is on average about 50% lower than the albedo at sites in the northern section of Atacama Desert (Fig. 2a). These differences were expected since the landscape in the southern Atacama Desert is greener (i.e. has more vegetation; see pictures in Fig. S3) than the northern Atacama Desert, which in turn makes the local albedo lower.

Healthy vegetation with abundant of chlorophyll reflects more NIR radiation and less UV and visible light^37^. For example, the albedo in the visible range of green grass is (about 0.05; Fig. S6a) at least 3 times lower than the average albedo that we measured in the Atacama Desert (about 0.17). For comparison, snow-covered surfaces exhibit in the visible range an albedo higher than 0.9 (Fig. S6b)^3^.

### Evaluation of MODIS-derived estimates

**Figure 3:**
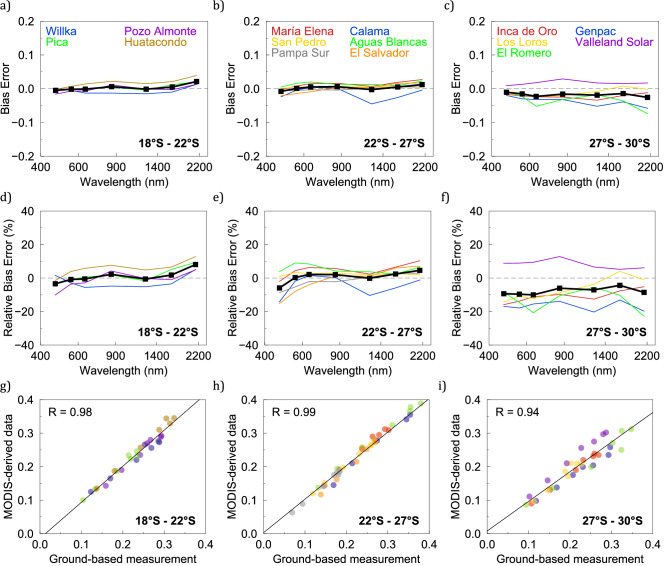
Comparison between MODIS-derived estimates of the blue-sky albedo and ground-based measurements of the albedo (taken as reference). (**a–c**) Bias error (thick line stands for the mean bias error), (**d–f**) Relative bias error (thick line stands for the relative mean bias error), (**g–i**) Scatter plots (correlation coefficients R are shown in the plots). For the comparisons, sites were clustered according to the latitude: from latitude 18° S to latitude 22° S (left-hand column), from latitude 22° S to latitude 27° S (central column) and from latitude 27° S to latitude 30° S (right-hand column). In plots (**a–f**), dots indicate the center of the MODIS bands. Plots were generated by using Python’s Matplotlib Library^44^.

Our ground-based measurements of the spectral albedo were compared with MODIS-derived estimates of the blue-sky albedo (Fig. 3). The spectral albedo was convolved with the spectral response functions for bands 1–7 of the MODIS sensor. Measurements taken at Aguila, Proyecto Gen, Pampa Norte and Vicuña were not considered in the comparison because they were taken at SZA higher than 40°, while measurements at Huara were not considered either because the closest MODIS data grid available in late September (during the campaign) was too distant (more than 3 km away; see Table 1).

Most of the satellite-derived estimates corresponding to each of the seven MODIS bands were in good agreement with our ground-based measurements. In the case of sites at latitudes lower than 27° S, bias errors of satellite data were generally lower than ± 0.03 (Fig. 3a,b) except in the case of Calama, within which satellite-derived estimates corresponding to Band 5 (1230–1250 nm) showed a slightly larger deviation. Bias errors of satellite data increased to ± 0.05 (Fig. 3c) in case of sites in the southernmost part of the Atacama Desert (27–30° S). These larger differences were expected since the landscape in the southern Atacama Desert is not only greener but also significantly less homogeneous than the landscape in the northern part. For example, the Elqui River Valley (around latitude 29° S) is fed from an Andean glacier that supports scattered vineyards and croplands^38^.

In the case of sites at latitudes lower than 27° S, the relative bias errors of MODIS-derived estimates were less than ± 5% (Fig. 3d-e). This can be considered a good correspondence since validation efforts elsewhere comparing the blue-sky albedo with tower-based albedo measurements have also shown that the bias of the 500 m MODIS operational albedo is generally within 5% of the field data^15^.

Satellite-derived estimates for sites in the southernmost part of the Atacama Desert (27–30° S) generally exhibited negative relative bias errors (up to about − 20%). These larger discrepancies can be attributed to the scattered vegetation in the region and the difference in the FOV between MODIS and our instrument. Although the sites selected for our ground-based measurements were relatively flat, level and homogeneous (Figs. S1–S3), differences between satellite estimates and ground-based measurements are expected to occur due to the different footprints. Limited by the input optics height, the circular footprint of our measurements (about 2 m in diameter) does not match the satellite grid (500 m × 500 m). While our measurements were carried out over a plant-free soil, satellite imagery shows that scattered vegetation in the southernmost part of the Atacama Desert (27–30° S) is likely inducing a considerable heterogeneity in the pixel in relation to the limited FOV of our instrument. As pointed out above, the albedo in the visible range of vegetation is substantially lower than albedo corresponding to the plant-free soil. This means that the scattered vegetation in the satellite FOV is likely lowering MODIS-derived estimates, which explains the mostly negative relative bias errors shown in Fig. 3f.

**Figure 4:**
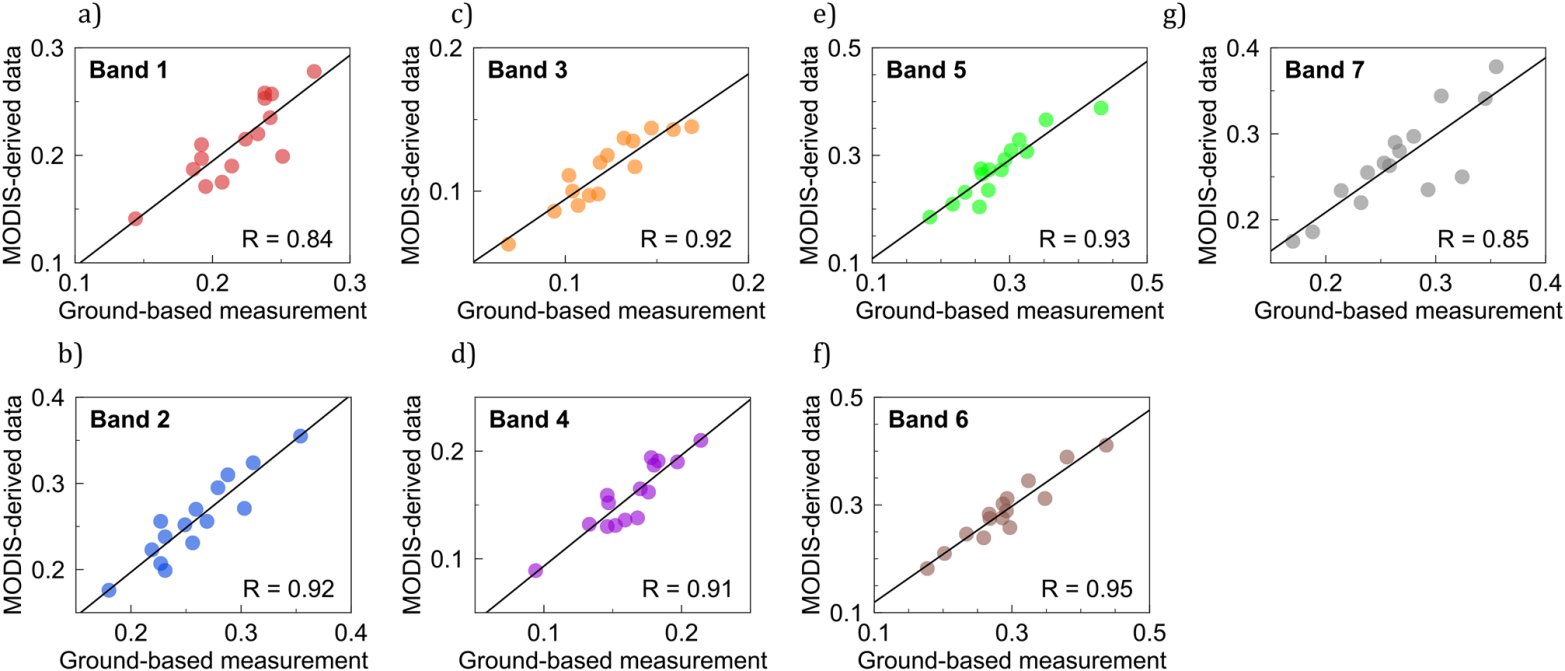
Scatter plots between MODIS-derived estimates of the blue-sky albedo and ground-based measurements of the albedo for the MODIS bands. (**a**) Band 1 (620–670 nm), (**b**) Band 2 (841–876 nm), (**c**) Band 3 (459–479 nm), (**d**) Band 4 (545–565 nm), (**e**) Band 5 (1230–1250 nm), (**f**) Band 6 (1628–1652 nm), and (**g**) Band 7 (2105–2155 nm). Each dot stands for the comparison at a measurement site. Correlation coefficients R are shown in the plots. Plots were generated by using Python’s Matplotlib Library^44^.

The correlation between the MODIS estimates and ground-based measurements of the albedo is generally high. In the case of sites at latitudes lower than 27° S, R-values were higher than 0.98 (Fig. 3g,h). The correlation coefficient slightly fell to 0.94 (Fig. 3i) in the case of sites in the southernmost part of the Atacama Desert (27–30° S). Again, the larger differences in the southern Atacama Desert are likely attributable to less homogeneous landscape of the southern Atacama Desert. Relatively high correlations between the MODIS estimates and ground-based measurements of the albedo were also found when examining individual MODIS bands (Fig. 4). R-values were generally higher than 0.91 except for Band 1 (620–670 nm) and for Band 7 (2105–2155 nm), within which the R-values were 0.84 and 0.85, respectively. The relatively lower signal-to-noise ratio of our measurements within the spectral range corresponding to these bands likely contributed to these lower correlations; the reflectance (and in turn the signal) was relatively low over the range 2105–2155 nm due to a water vapor absorption band.

Differences between MODIS estimates and ground-based measurements of the albedo can also arise from scattering process in the atmosphere. In particular, over bright surfaces, it is a challenging task to distinguish between the contribution of surface reflectance and aerosol scattering and multiple reflections between the two layers (the surface and the scattering atmosphere). Although aerosols tend to be abundant in deserts due to dry and arid conditions, satellite-retrieved estimates of the Aerosol Optical Depth (AOD) over the Atacama Desert are on average consistently lower than 0.1^39^. These low AOD values roughly agree with ground-based measurements carried out at the Paranal Observatory (2635 m AMSL, 24° 37′ S, 70° 24′ W)^40^. For comparison, the AOD at 500 nm is typically higher than 0.15 at sites in North Africa^41^, while the AOD in the visible range measured at desert sites in northern China is on average about 0.3^42^.

In the Supplementary Material (Table S1), for each MODIS band, we present the correlations between the relative bias error of MODIS-derived estimates and the following parameters: the SZA at the moment of the measurement, the distance between the measurement site and the center of the closest MODIS data grid, and the latitude and elevation of the measurement site. These correlations may suggest potential issues that affect the retrieval algorithm. We found weak correlations between MODIS biases and the SZA (Table S1), which suggests a limited anisotropy of the surface reflective properties of the Atacama Desert. Surfaces have directional properties such that their brightening may change with the angle of illumination (SZA)^43^. However, this effect is not apparent in our comparisons that nevertheless were constrained to a relatively narrow SZA range (17–36°). The correlations between MODIS biases and the distance to the center of the closest MODIS data grid were insignificant (Table S1). Similarly, no significant correlation was found between MODIS biases and the elevation of the measurement site. We found slightly stronger correlations between MODIS biases and latitude of the measurement site (Table S1). R-values ranged from + 0.33 to + 0.47 for MODIS bands 1 through 3 (in the visible range) that are more sensitive to the vegetation. *R*-values were predominantly positive, which indicates that MODIS biases tend to increase southward.

### Comparisons with other arid and semi-arid regions

**Figure 5:**
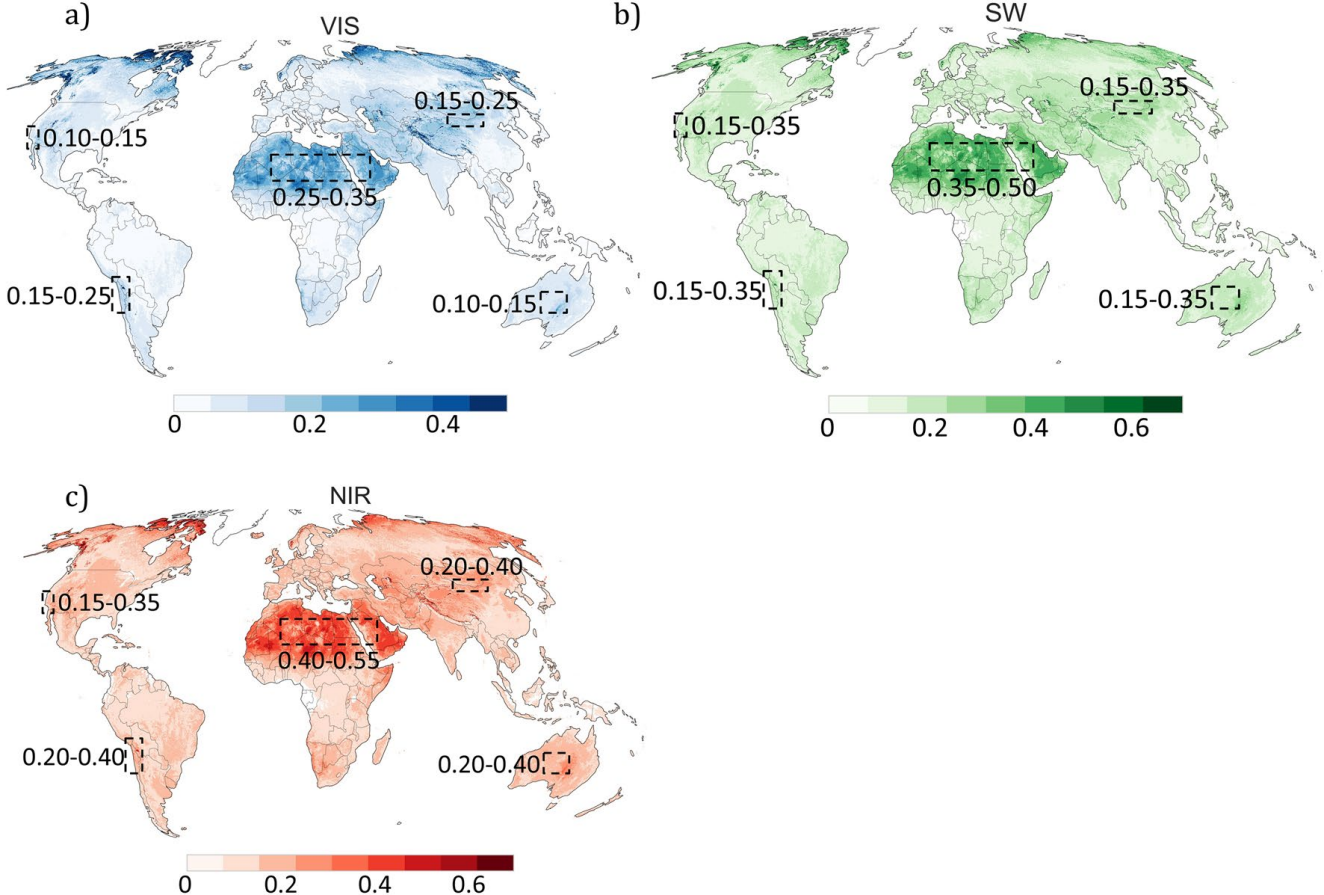
Satellite-derived estimates of the white-sky albedo averaged over the period 2011–2020 for the following broadbands: (**a**) Visible (VIS), (**b**) Shortwave (SW), and (**c**) Near infrared (NIR). Plots were generated using Python’s Matplotlib library^44^ with data from the Moderate Resolution Imaging Spectroradiometer (MODIS) MCD43A3 Version 6 Albedo Model dataset^24^.

Our measurements confirm that the albedo of the Atacama Desert, especially at latitudes lower than 27° S, is relatively high, which is particularly useful for bifacial solar PV technologies. It is worth comparing the albedo of the Atacama Desert with the albedo of other regions of rapid solar PV energy growth. MODIS data averaged over the period 2011–2020 show that in general arid or semi-arid regions exhibit high albedo (Fig. 5).

In the shortwave (SW) range, the highest albedo is expected to occur in the Sahara Desert and the Arabian Peninsula. In these regions, MODIS estimates of the SW albedo are on average higher than 0.35. In other arid or semi-arid regions (Central Australia, Gobi Desert, Southern California and the Atacama Desert), MODIS estimates of the SW albedo are significantly lower (ranging from 0.15 to 0.35). In the visible (VIS) range (the most important for silicon-based PV technologies), the highest albedo is also expected in the Sahara Desert and the Arabian Peninsula. MODIS estimates show that the VIS albedo is generally higher than 0.25 for these regions. Although it can be higher at specific locations, MODIS estimates of the VIS albedo generally range from 0.15 to 0.25 in the case of Atacama Desert and the Gobi Desert, while they are generally lower than 0.15 in the case of Central Australia and Southern California.

## Discussion and conclusions

The albedo determines the fraction of the incoming solar irradiance reflected by the surface. Here, we report on the first quality-controlled ground-based measurements of the spectral albedo (350–2200 nm) in the Atacama Desert. The measurements were carried out under cloudless conditions at 20 sites across a north–south transect of approximately 1300 km, from latitude 18° S to latitude 30° S; site elevation ranged from 485 m AMSL to 2583 m AMSL.

We found that the albedo of the Atacama Desert ranges from about 0.1 to about 0.25 in the visible part of the spectrum. We also found that, likely due to the more abundant (although scattered) vegetation, the albedo in the southernmost part of the Atacama Desert (27–30° S) is slightly lower than the albedo in the northernmost section of the desert (18–22° S).

Our ground-based measurements were used as a reference to test MODIS-derived estimates of the blue-sky albedo for the Atacama Desert. We found that at latitudes lower than 27° S, the relative mean bias error of MODIS-derived estimates is within ± 5% of the ground-based measurements. We also found that in the southernmost part of the Atacama Desert (27–30° S), MODIS data exhibit a relative mean bias error of about − 8% that can be attributed to the FOV mismatch between MODIS and our instrument. Due to their larger footprint, satellite estimates are likely influenced by the scattered vegetation, which is more abundant in the less arid southernmost part of the Atacama Desert.

The correlation between MODIS estimates and ground-based measurements of the albedo was found to be generally high, and the retrieval algorithm does not appear to be significantly influenced by the anisotropy of the surface reflective properties, the angular configuration (illumination and viewing angles), or scattering process in the atmosphere. Despite our efforts, validation of satellite product requires long-term measurements. While surface albedo is routinely measured at a number of research sites, the measurement network is sparse and it does not include sites in the Atacama Desert. Long-term records of surface albedos are still required in the Atacama Desert to support climate, biogeochemical, hydrological, and solar energy studies.

Both MODIS-derived data and ground-based measurements show that the albedo at some bright spots in the Atacama Desert may be high enough for considerably boosting the performance of bifacial PV technologies. The enhanced surface-reflected irradiance of bright arid and semi-arid regions can improve the performance of bifacial PV modules leading to a “bifacial gain” (i.e., an extra energy yield) relative to identically oriented and tilted monofacial modules. According to the formulation proposed by Sun et al. (2018)^12^, the bifacial gain for ground-mounted north-oriented bifacial solar modules at our measurement sites would range from about 6% at Pampa Sur to about 12% at Huara (see Table S2).

Comparisons with other regions of rapid solar PV energy growth show that, in the visible part of the spectrum (the most important for silicon-based PV technologies), the albedo of the Atacama Desert lies in the same range as the albedo of the Gobi Desert and is on average higher than the albedo of Central Australia and Southern California. However, it falls short compared with the albedo of the Sahara Desert and the Arabian Peninsula, the brightest regions on Earth outside the poles.

## Data Availability

Remote sensing estimates from the Moderate Resolution Imaging Spectroradiometer (MODIS) MCD43A3 Version 6 Albedo Model dataset are available from: https://lpdaac.usgs.gov/products/mcd43a3v006/. Ground-based measurements used in this study are available from the corresponding author.

## Supplementary﻿ Information

Supplementary Information.
