# Supplementary material for: Evaluation of MODIS-derived estimates of the albedo over the Atacama Desert using ground-based spectral measurements
Source: Sci Rep. 2021 Oct 6;11:19822. doi: 10.1038/s41598-021-98622-4 (PMC8494836; doi:10.1038/s41598-021-98622-4)
Supplement: Supplementary file 1 — Supplementary Information. [file 41598_2021_98622_MOESM1_ESM.pdf]

# Supplementary Material

## Evaluation of MODIS-derived estimates of the albedo over the Atacama Desert using ground-based spectral measurements

Raúl R. Cordero[1], Sarah Feron[1,2,*], Edgardo Sepúlveda[1], Alessandro Damiani[3], Juan M. Carrera[4], Jose Jorquera[1], Juan A. Alfonso[4], Rosalino Fuenzalida[5], Miguel Rivas[6], Shelley MacDonell[7], Gunther Seckmeyer[8], Chenghao Wang[9], Zutao Ouyang[9], Stef Lhermitte[10]

1 Universidad de Santiago de Chile. Av. Bernardo O'Higgins 3363, Santiago, Chile.
2 University of Groningen, Wirdumerdijk 34, 8911 CE, Leeuwarden, Netherlands.
3 Center for Environmental Remote Sensing, Chiba University, 1-33 Yayoicho, Inage Ward, Chiba, 263-8522, Japan
4 Instituto Venezolano de Investigaciones Cientificas (IVIC), Apartado 20632, Caracas, 20632, Venezuela.
5 Universidad Arturo Prat, Avenida Arturo Prat 2120, Casilla 121, Iquique, Chile.
6 Universidad de Tarapacá, Avenida General Velásquez 1775, Arica, Chile.
7 Centro de Estudios Avanzados en Zonas Aridas (CEAZA), La Serena, Chile.
8 Leibniz Universität Hannover, Herrenhauser Strasse 2, Hannover, Germany.
9 Department of Earth System Science, Stanford University, Stanford, CA, 94305, USA.
10 Department of Geoscience and Remote Sensing, Delft University of Technology, Delft, The Netherlands

* Corresponding Author
Sarah Feron
s.c.feron@rug.nl
University of Groningen, Netherlands,

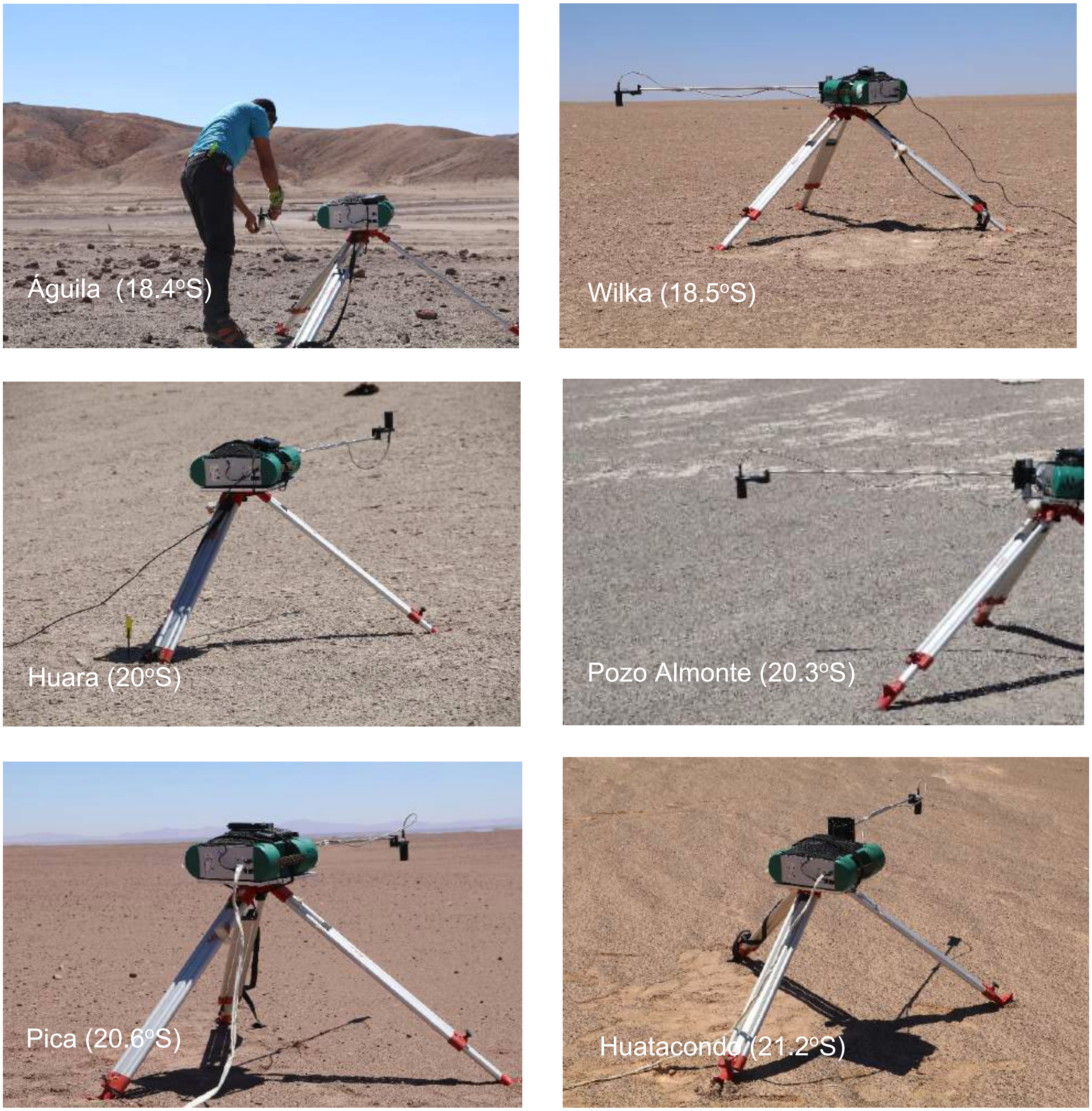


**Fig. S1.**
Measurement sites from latitude 18°S to latitude 22°S. Photographs were taken by the authors (J.J. and J.M.C).

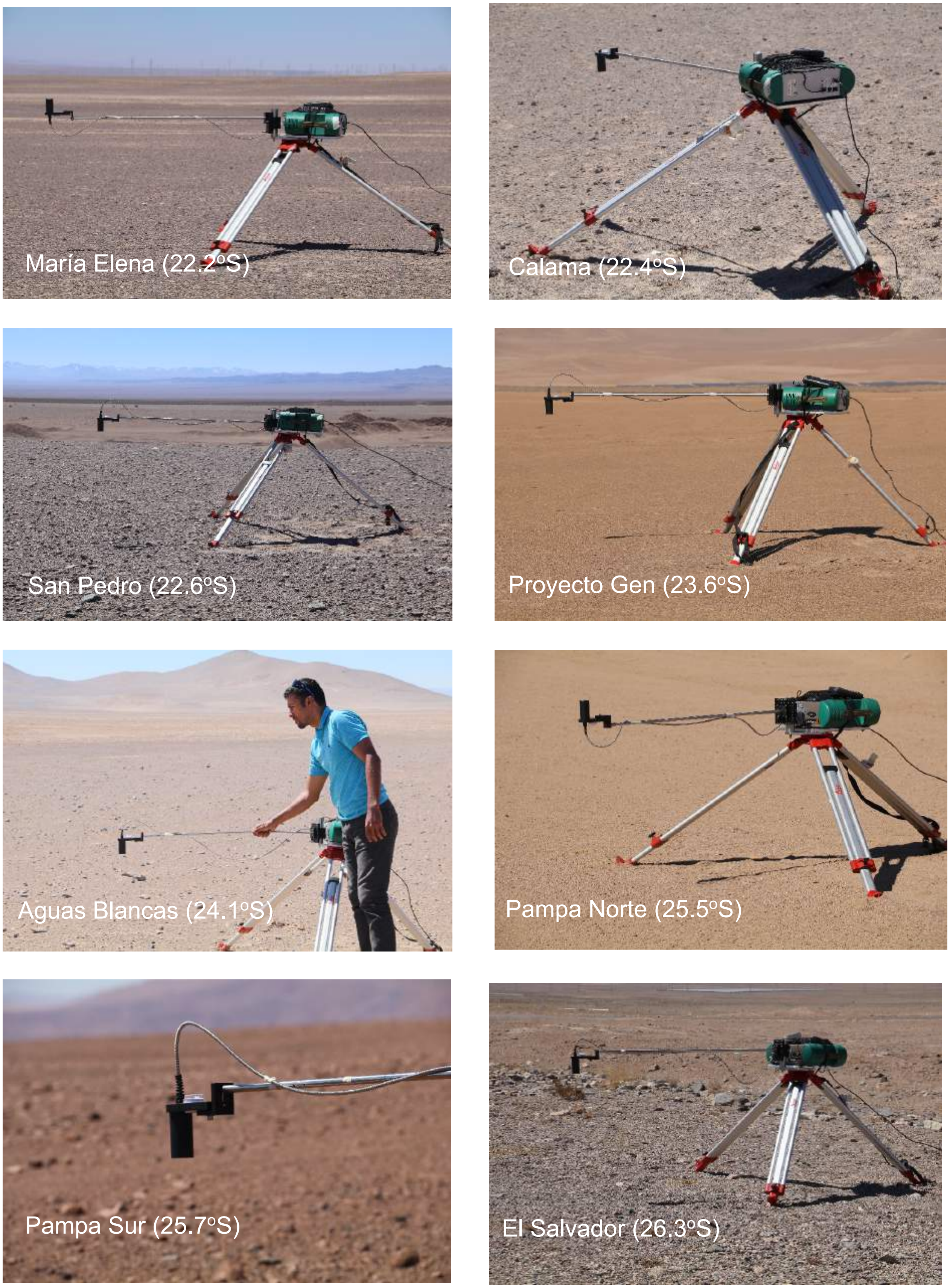


**Fig. S2.**
Measurement sites from latitude 22°S to latitude 27°S. Photographs were taken by the authors (J.J. and J.M.C).

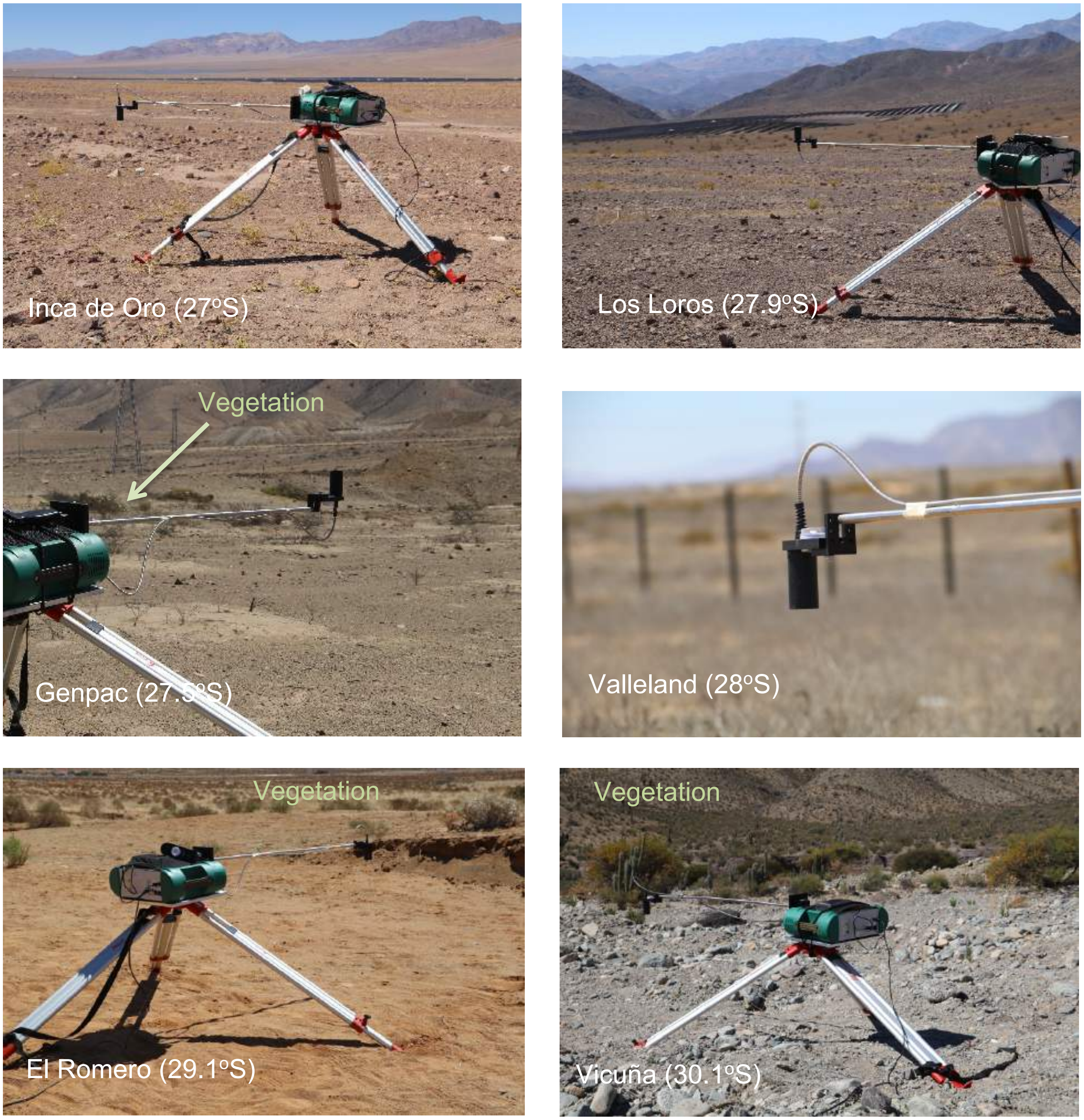


**Fig. S3.**
Measurement sites from latitude 27°S to latitude 30°S. Photographs were taken by the authors (J.J. and J.M.C).

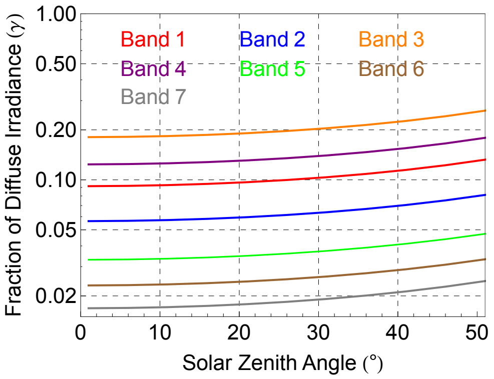


**Fig. S4.**
Fraction of diffuse irradiance computed for different solar zenith angles by using the UVSPEC radiative transfer model under the typical conditions of the Atacama Desert for the following MODIS bands:

Band 1 (620-670 nm),
Band 2 (841-876 nm),
Band 3 (459-479 nm),
Band 4 (545-565 nm),
Band 5 (1230-1250 nm),
Band 6 (1628-1652 nm), and
Band 7 (2105-2155 nm).

The plot was generated using Python's Matplotlib library[44].

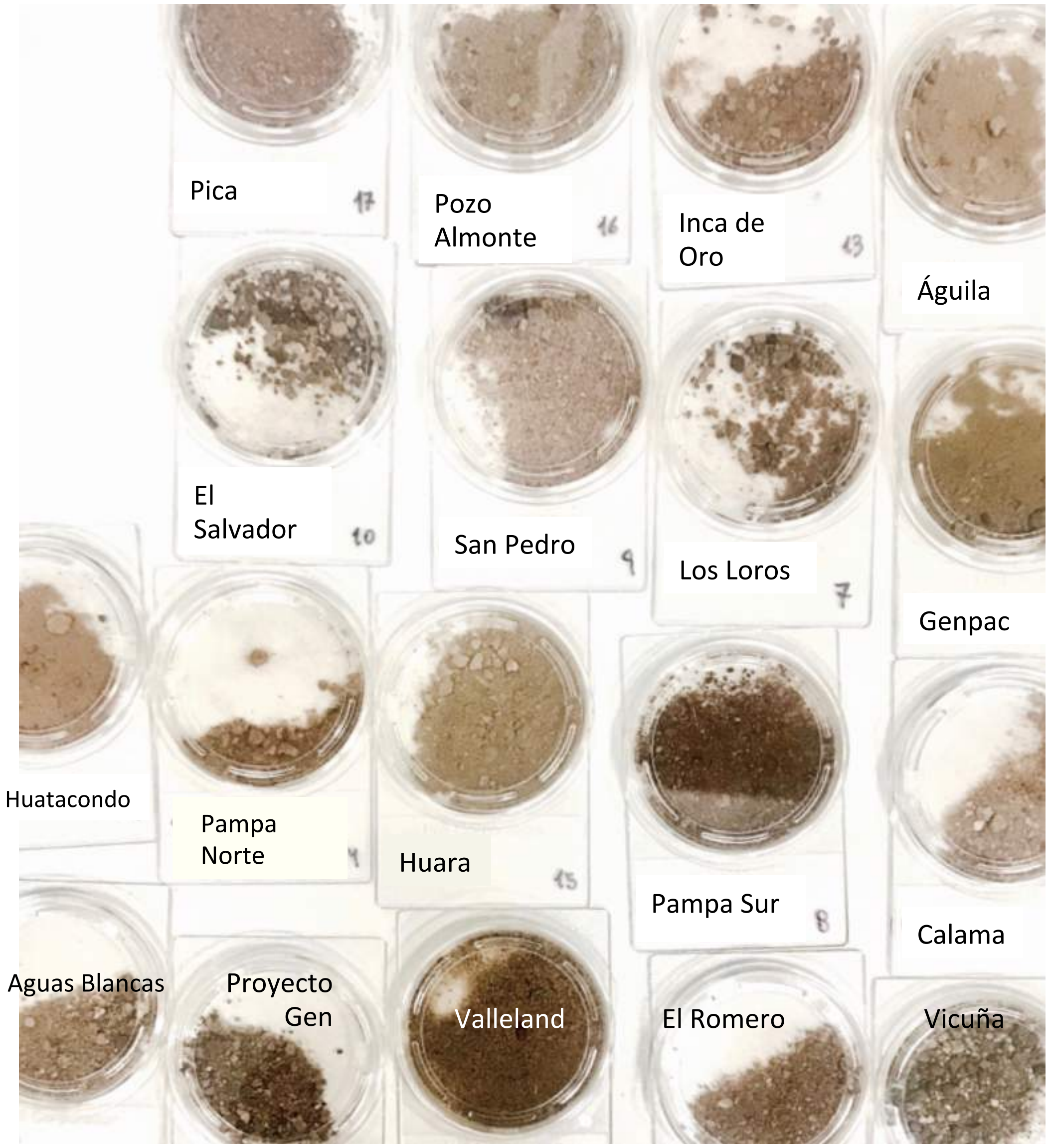


**Fig. S5.**
Dust samples taken from the surface at the measurement sites (samples from Wilka and María Elena are missing). Photograph was taken by the author (R.R.C.).

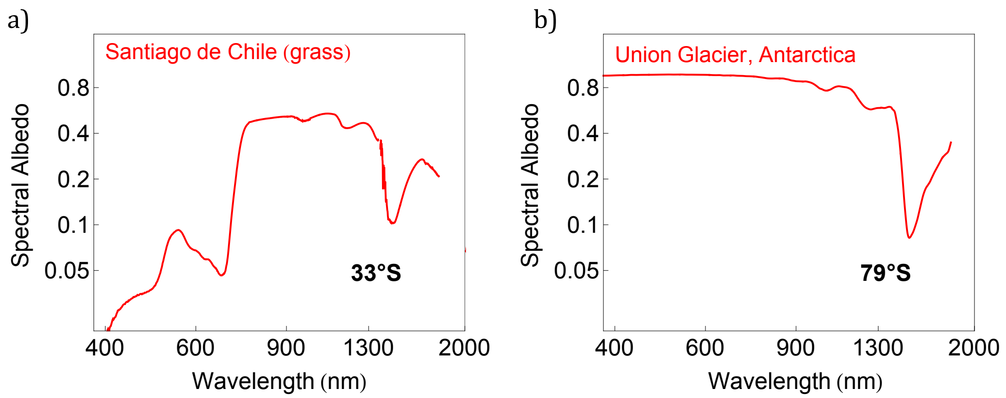


**Fig. S6.**
Ground-based measurements of the spectral albedo carried out in:
a) Santiago de Chile in April 2020 and
b) Union Glacier (Antarctica) in December 2015.
The measurements were carried out by using the same instrument and methods applied in the case of the measurement in the Atacama Desert. The measurement in Santiago was carried out over a surface covered by green grass while the measurement at Union Glacier was carried out over a glacier covered by several meters of snow. Antarctic albedo in the vast interior of Antarctica is normally higher than 0.9 in the visible range (400-700 nm).
Plot was generated by using Python's Matplotlib Library [44].

**Table S1**. Correlation coefficients between the relative bias error of the MODIS-derived estimates of the blue-sky albedo (relative to the ground-based measurements of the albedo) and the SZA (at the moment of the measurement), the distance between the measurement site and the center of the closest MODIS data grid, and the latitude and elevation of the measurement site.

| Bands | SZA | Distance | Latitude | Elevation |
|---|---|---|---|---|
| B1 | -0.17 | -0.17 | 0.47 | -0.06 |
| B2 | -0.18 | -0.18 | 0.40 | -0.11 |
| B3 | -0.17 | -0.09 | 0.33 | -0.37 |
| B4 | -0.15 | -0.23 | 0.47 | -0.12 |
| B5 | 0.09 | -0.30 | 0.23 | -0.14 |
| B6 | 0.04 | -0.14 | 0.28 | -0.03 |
| B7 | -0.27 | -0.09 | 0.61 | -0.04 |

**Table S2**. Bifacial gain of bifacial solar modules at the measurement sites. These estimations were conducted according to the formulation proposed by Sun et al. (2018)[12], which is available as an online simulation tool, i.e., Purdue University Bifacial Module Calculator[45]. For all north-oriented modules, we assumed a front-side efficiency of 18%, a temperature coefficient of 0.41%/ºC, a constant heat transfer component of 22.7 W/m²/ºC and a convective heat transfer component of 6.8 W.s/m³/ºC. For the rest of the parameters required for the estimations, we assumed the values indicated in the Table.

| Site | Latitude (°) | Longitude (°) | Elevation (m) / Height (m) | Bifaciality | Tilt Angle (°) | Albedo (VIS) | Bifacial Gain (%) (Annual Mean) |
|---|---|---|---|---|---|---|---|
| Águila | -18.4429 | -69.8929 | 1/1 | 0.8 | 20 | 0.14 | 7.7 |
| Wilka | -18.4937 | -70.1126 | 1/1 | 0.8 | 20 | 0.15 | 8.2 |
| Huara | -19.9937 | -69.7528 | 1/1 | 0.8 | 20 | 0.25 | 12.4 |
| Pozo Almonte | -20.2655 | -69.7508 | 1/1 | 0.8 | 20 | 0.17 | 9.1 |
| Pica | -20.5668 | -69.4733 | 1/1 | 0.8 | 20 | 0.13 | 7.2 |
| Huatacondo | -21.1721 | -69.5597 | 1/1 | 0.8 | 20 | 0.15 | 8.1 |
| María Elena | -22.2207 | -69.5965 | 1/1 | 0.8 | 23 | 0.16 | 8.7 |
| Calama | -22.4302 | -68.8725 | 1/1 | 0.8 | 23 | 0.18 | 9.4 |
| San Pedro | -22.5762 | -68.7030 | 1/1 | 0.8 | 23 | 0.14 | 7.5 |
| Proyecto Gen | -23.5643 | -70.2297 | 1/1 | 0.8 | 23 | 0.15 | 8.4 |
| Aguas Blancas | -24.0897 | -69.9284 | 1/1 | 0.8 | 25 | 0.16 | 8.9 |
| Pampa Norte | -25.5121 | -70.1761 | 1/1 | 0.8 | 25 | 0.18 | 9.8 |
| Pampa Sur | -25.7451 | -70.3683 | 1/1 | 0.8 | 25 | 0.10 | 6.2 |
| El Salvador | -26.2864 | -69.6482 | 1/1 | 0.8 | 25 | 0.17 | 9.0 |
| Inca de Oro | -27.0346 | -69.9107 | 1/1 | 0.8 | 28 | 0.15 | 8.4 |
| Genpac | -27.4886 | -70.3798 | 1/1 | 0.8 | 28 | 0.16 | 9.1 |
| Los Loros | -27.8618 | -70.1832 | 1/1 | 0.8 | 28 | 0.15 | 8.5 |
| Valleland | -28.1214 | -70.6103 | 1/1 | 0.8 | 28 | 0.15 | 8.9 |
| El Romero | -29.1274 | -70.9282 | 1/1 | 0.8 | 30 | 0.14 | 8.3 |
| Vicuña | -30.0617 | -70.7660 | 1/1 | 0.8 | 30 | 0.16 | 9.2 |
